# Three-factor structure for Epistemic Belief Inventory: A cross-validation study

**DOI:** 10.1371/journal.pone.0173295

**Published:** 2017-03-09

**Authors:** Francisco Leal-Soto, Rodrigo Ferrer-Urbina

**Affiliations:** 1 Department of Social Sciences, University of Tarapacá at Iquique, Iquique, Chile; 2 Center for Research in Inclusive Education, Viña del Mar, Chile; 3 Psychology and Philosophy School, University of Tarapacá at Arica, Arica, Chile; Institut Català de Paleoecologia Humana i Evolució Social (IPHES), SPAIN

## Abstract

Research on epistemic beliefs has been hampered by lack of validated models and measurement instruments. The most widely used instrument is the Epistemological Questionnaire, which has been criticized for validity, and it has been proposed a new instrument based in the Epistemological Questionnaire: the Epistemic Belief Inventory. The Spanish-language version of Epistemic Belief Inventory was applied to 1,785 Chilean high school students. Exploratory and confirmatory factor analyses in independent subsamples were performed. A three factor structure emerged and was confirmed. Reliability was comparable to other studies, and the factor structure was invariant among randomized subsamples. The structure that was found does not replicate the one proposed originally, but results are interpreted in light of embedded systemic model of epistemological beliefs.

## Introduction

Epistemic beliefs are beliefs about knowledge and its acquisition. They have an important role in various processes related to learning, self-regulation and academic achievement, as different authors have highlighted and empirical evidence supports [1–6]. In addition, there is evidence that epistemic beliefs can be modified with specific interventions [7–9], so change naïve epistemic beliefs could be a way to optimize learning processes.

Despite there are various models of epistemic beliefs emphasizing some different aspects, as the evolution of thinking process about knowledge and knowing [10], [11] the differences between women and men in thinking about knowledge [12], [13], the role of epistemic perspective in decision making [14], the attitude or disposition of teachers regarding knowledge and knowing process [15], or the resources character of epistemic beliefs [16], the model most commonly used in research has been proposed by Marlene Schommer [17], [18]. For her, epistemic beliefs are a set of more or less independent dimensions, whose development does not necessarily follow a homogeneous sequence but evolves from a naive dualist position on knowledge and learning to a relativistic and sophisticated position. Based on this model, she developed the Epistemological Questionnaire (EQ), from which she set five dimensions: knowledge structure, stability or certainty of knowledge, source of knowledge, learning control and speed of learning [17]. However, the factor structure of the EQ has been only partially and inconsistently found in empirical studies, and their reliability has been unsatisfactory [19], leading some researchers to propose the Epistemic Belief Inventory (EBI) [20], [21] as a way to overcome the original psychometric weaknesses of the EQ [22]. The EBI maintains some of the original items of EQ and adds new items to form five dimensions equivalent to Schommer’s model: Omniscient Authority (source of knowledge), Quick Learning (learning speed), Simple Knowledge (knowledge structure) and Innate Ability (learning control).

Despite intense research use, these instruments have shown great volatility in their factorial structure and other psychometric indicators, both in the United States and other cultural contexts [23]. For example, in the EQ, it has been replicated four of the five dimensions (knowledge structure and stability, control and speed of learning) for Norwegian students [24], but it has been reported only two dimensions (single learning and structure of knowledge) in Filipino preservice teachers [25]. With the EBI something similar happens: it has been found the dimensions of true knowledge, innate ability and quick learning in Korean students, [26], but in Turkish education students the dimensions of rapid learning, innate ability and certain knowledge were described [27]. It has been found the proposed five factors in a sample of Singaporean teachers, but the items that comprise each factor in their sample are different than those originally proposed [28]; while, on Chinese undergraduates, the proposed five factors structure achieved good indicators of adjustment, but retaining only 25 items, after unsuccessfully trying two other models [29].

The mixed and unstable nature of these findings has been highlighted every time the factorial structure of epistemic beliefs has been revised; for example, Hofer and Sinatra [30] explicit it when closing a special issue on the subject, and Schraw, following a conceptual and empirical review, concludes that the factor structure of the instruments for evaluating epistemic beliefs has been very dependent on the sample [31].

The internal consistency of the dimensions has also been problematic. In the case of the EBI, the consistency varies according to the study and the sample from Cronbach’s α values as low as .32 [32] until suitable values such as .88 [33]. Meanwhile, DeBacker et al. have noted that the samples in most of the studies on the EBI have been "modest" in size, a situation that could contribute to these poor results [23]; however, the same work of DeBacker et al., which included more than 300 participants, only reached a reliability level between α = .47 and α = .67.Subsequent work has included larger samples without better results. For example, it has been reported consistencies between α = .41 and α = .75 with a sample of 1,876 participants, and any factor model failed to reach acceptable adjustment indicators [34]. Something similar was reported in [35], whose consistencies ranged from α = .36 and α = .75 with a sample of 282 participants.

A work that deserves special mention is Paetcher et al. [36]. They developed a new tool for use in Germany, called the Oldenburg Epistemic Beliefs Inventory. They used the EBI as their basis, but they added new items and considered only four of the five original dimensions (source of knowledge, knowledge structure, learning control and speed of learning). With this version of the instrument, the proposed four-factor structure emerged on a first sample with exploratory techniques and was replicated on a second sample using confirmatory factor analysis techniques. However, this study uses a different structure and only four factors, and it persists with poor reliability levels (ranging α = 0.50 to α = 0.76).

The situation in Latin-America or Spanish-spoken populations is no different. With the EQ we found five studies, of which only three reported psychometric indicators and factor structures. In Venezuelan medical students four dimensions has been found (simple knowledge, true knowledge, learning control and speed of learning) [37]; a structure that recognizes learning control, speed of learning, knowledge structure and certainty of knowledge dimensions has been described in Spanish university students [38]; and it has been reported 12 factors, but chose not to group items by factors but to obtain scores directly proposed by the model, in Chilean student teachers [39]. None of these three works reports reliability by dimensions, but they report only a full scale Cronbach´s Alpha indicator, which is not interpretable in a multidimensional scale [40], ranging from α = .66 to α = .69.

With the EBI we found only two studies: one in Chilean student teachers reported in [41], and one in Argentinean high school students [42]. In the first one, a five-dimensional structure was found, but only three corresponded to the original dimensions; the other two were called *omniscient authority* (certain knowledge) and *futility of the effort*. In the second study, five dimensions that corresponded to the proposals were found, but the source dimension of knowledge was discarded due to its low reliability. The reliabilities for the retained dimensions ranged from α = .33 and α = .63. Two other works that explore the epistemic beliefs of teachers and student teachers [43], [44] used an EBI-based instrument [45], to which the authors attributed good psychometric properties but they did not provide details. In Table 1 you can see results of some of the studies with the EQ and the EBI with information about the reported factorial structure.

**Table 1:** Results regarding the confirmation of the five-factor structure proposed for the EQ and the EBI in various studies.

Publication year	Authors	Instrument	Confirms five- factor structure
1990 [17]	Schommer	EQ	No
1992 [46]	Schommer, Crouse & Rhodes	EQ	No
1993 [47]	Jehng, Johnson & Anderson	EQ	Yes
1993 [48]	Schommer	EQ	No
1994 [49]	Schommer & Dunnell	EQ	No
1995 [50]	Quian & Alverman	EQ	No
1995 [21]	Schraw, Dunkle & Bendixen	EBI	Yes
1998 [51]	Bendixen, Schraw & Dunkle	EBI	No
2002 [20]	Schraw, Bendixen & Dunkle	EQ	No
2002 [20]	Schraw, Bendixen & Dunkle	EBI	Yes
2003 [52]	Nietfeld & Enders	EBI	Yes
2003 [53]	Nussbaum & Bendixen	EBI	No
2004 [41]	Mejía & Palma (in Leal-Soto, 2008)	EBI*	No
2005 [54]	Ravindran, Greene & DeBacker	EBI	Yes
2006 [55]	Bell	EBI	No
2007 [56]	Hardré, Crowson, Ly & Xye	EBI	Yes
2008 [25]	Bernardo	EQ	No
2008 [33]	Müller, Rebmann & Liebsch	EBI	No
2009 [37]	Sánchez	EQ*	No
2010 [32]	Laster	EBI	No
2010 [42]	Martínez, Montero & Pedrosa	EBI*	No
2010 [26]	So, Lee, Roh & Lee	EBI	No
2011 [34]	Teo & Chai	EBI	No
2012 [27]	Cam, Topeu, Sulun, Guven & Arabacioglu	EBI	No
2012 [28]	Teo & Chai	EBI	No
2012 [38]	De Juanas & Beltrán	EQ*	No
2012 [39]	Schommer-Aikins, Beuchat-Reichardt & Hernández-Piña	EQ*	No
2013 [57]	Taha & El-Habbal	EBI	Yes
2013 [29]	Wang, Zhang, Zhang & Hou	EBI	Yes
2013 [35]	Welch & Ray	EBI	No

* In Spanish-language and Spanish or Latin-American sample.

Another concern is the role of gender in epistemic beliefs and their measurement, which is fairly unclear. Evidence on gender differences in epistemic beliefs are contradictory [23], but more importantly, studies have not reported confirmation that epistemic beliefs are manifested in the same way, that is, if the factorial structure postulated is similar for both genders, which is another weakness of these instruments.

Other fact to note is that, outside the United States, most of the studies have been conducted with college students, with very few dedicated to high school students; and of these studies, only one was conducted in a Spanish-speaking context [42]. For this reason, the possibility of contrasting the reported international literature on the basis of studies conducted mainly with these instruments or contributing to the expansion of knowledge of the relationship between epistemic beliefs and learning processes in high school students is limited in Spanish-speaking contexts.

In summary, the epistemological beliefs model of Marlene Schommer is widely used in research, but instruments used to measure it, both EQ and EBI, have several problems that hinder comparison and integration of research results. In this paper, we proposed to bring some clarity regarding the factor structure and internal consistency of the EBI, because it was intended to improve the EQ, and have the advantage of terseness; and to explore the possible influence of gender on the factor structure, what has been neglected in previous research. Moreover, we do this in Chilean high-school students, a Latin-American and Spanish-speaking population. This knowledge would allow a more precise use of the EBI in the study of the influence of personal epistemology on learning processes and conceptual change in Spanish-speaking contexts, contributing to a more precise assessment of Schommer’s model of epistemological beliefs.

## Method

The study reported in this paper was approved as part of research project FONDECYT 1110722 by Ethics Committee of Fondo Nacional de Desarrollo Científico y Tecnológico and by Ethics and Bioethic Committee of University of Tarapacá.

### Participants

Participants were 1,785 high school students (from 7th to 12th grade) from public schools and private schools with public funding from the cities of Iquique and Arica, in northern Chile. Of these, 49.8% were female, and ages ranged from 12 to 19 years. Although there was more availability of intermediate classes than elementary or advanced ones, all high school classes were represented, from freshmen to senior; however, the distribution by age and grade was not homogeneous. This sample was divided randomly into two subsamples of approximately 60% and 40% respectively; the first sample had 1,039 participants (subsample 1), and the other had 746 participants (subsample 2). The first subsample was used for exploratory analyses, and the second, for confirmatory analyses.

### Materials and procedure

#### Instruments

We use a local Spanish language adaptation of the Epistemic Belief Inventory (EBI) developed by Schraw et al. [20]. It is an instrument composed of 28 five-point Likert scale items, each one consisting of a statement regarding epistemic beliefs and following the model of five dimensions of Schommer-Aikins [18] described in the introduction. The dimensions, as described in [20], are: 1, Omniscient Authority (e.g., "People shouldn't question authority); 2, Certain Knowledge (e.g., "What is true today will be true tomorrow"); 3, Quick Learning (e.g., "Working on a problem with no quick solution is a waste of time"); 4, Simple Knowledge (e.g., Too many theories just complicate things"); 5, Innate Ability (e.g., "Smart people are born that way").The principal author conducted a translation from English into Spanish, which was back-translated into English by an independent English-speaker to ensure equivalence.

#### Procedure

The instrument was administered at schools during a regular class period with permission of principals, as part of a broader research program in which students and teachers participated. Participation was voluntary and with the consent of the students’ parents.

#### Analysis of data

First, random subsample 1 (n = 1,039) was used to establish an initial model. In order to maximize comparability with other studies, we used a principal component analysis, as previous studies on the subject have done [20], [39], [42]. It should be noted that the principal component extraction method is not a factorial estimate [58], albeit it is a common misconception to report it as factor analysis [59], [60], [61], [62]. However, in practice, principal components analysis is usually equivalent to factorial estimates [63]; a factor analysis was conducted in parallel with the principal component analysis to assess whether the equivalence enable us to interpret the principal component analysis as the right solution. According to the Kolmogorov-Smirnov test, the null hypothesis of normality in all items was refused (*p* < .01), ruling out the existence of multivariate normality; the factorial analysis was performed using the generalized least squares method of extraction, which is robust in this scenario [64], [65]. The result of the exploratory factor analysis (see S1 Table) was equivalent to the final structure obtained by the method of main components (Table 2), which is why we settled on the principal component analysis method, assuming that it is a good approximation of the exploratory factor analysis in this scenario.

**Table 2:** Loading for components and internal consistency for proposed solution.

Item	Component		
	1	2	3
1. La mayoría de las cosas valiosas son fáciles de entender (Most things worth knowing are easy to understand).	.025	.097	**.597**
2. Lo que se considera verdadero es una cuestión de opinión (What is true is a matter of opinion).	-.078	.001	**.679**
3. Los estudiantes que aprenden rápido son los más exitosos (Students who learn things quickly are the most successful).	**.565**	.222	.142
4. La gente debería obedecer siempre la ley (People should always obey the law).	.057	.**769**	.015
5. La capacidad intelectual de las personas está determinada desde el nacimiento (People’s intelectual potential is fixed at birth).	**.593**	.125	-.020
8. Los estudiantes realmente inteligentes no necesitan esforzarse para que les vaya bien en la escuela (Really smart students don´t have to work as hard to do well in school).	**.549**	-.102	.136
9. Si alguien se esfuerza demasiado para entender un problema, probablemente terminará confundiéndose (If a person tries too hard to understand a problem, they will most likely end up being confused).	**.430**	-.100	.229
11. Con frecuencia, las mejores ideas son las más simples (The best ideas are often the most simple).	.105	.074	**.552**
14. Qué tan bien te vaya en la escuela depende de qué tan listo seas (How well you do in school depends on how smart you are).	**.575**	.142	-.018
15. Si no aprendes algo rápidamente, nunca lo aprenderás (If you don’t learn somethin(g quickly, you won’t ever learn it).	**.676**	-.055	-.038
17. Las cosas son más simples de lo que la mayoría de los profesores quisiera hacernos creer (Things are simpler tan most professors would have you believe).	.140	.002	**.469**
20. Si no entiendes un capítulo la primera vez que lo lees, volver atrás no ayudará (If you haven’t understood a chapter the first time through, going back over it won´t help).	**.524**	-.140	-.188
22. Mientras más sabes de algo, más queda por saber (The more you know about a topic, the more there is to know).	-.171	.071	**.476**
24. Las personas habilidosas nacieron así (Smart people are born that way).	**.539**	.154	.007
25. Cuando alguna autoridad me dice qué hacer, generalmente lo hago (When someone in authority tells me what to do, I ussually do it).	-.071	**.747**	.170
26. Las personas no deberían cuestionar la autoridad (People shouldn’t question authority).	.140	**.693**	.058
27. Trabajar en un problema que no se soluciona rápido es una pérdida de tiempo (Working on a problem with no quick solution is a waste of time).	**.625**	.013	-.053
Internal consistency (Cronbach’s α)	.74	.63	.49

Note. Spanish-language items are numbered in correspondence with [20], p. 275. Loadings of retained items in each component are bold.

Subsequently, the resulting model was tested using a confirmatory factor analysis with random subsample 2 (n = 746) to prevent the effects of capitalization on chance [64]. Additionally, we compute the fit indexes of two alternative models with five factors structures: the original model of Schraw, Bendixen and Dunkle [20] and the model in Chileans university students reported in [41]. In addition, we proceeded to perform three multigroup confirmatory factor analyses: 1) two randomized subgroups to assess the stability of the estimate; 2) gender; and 3) city of residence (Arica and Iquique). All the estimations was made following recommendations on the factorial treatment of ordinal variables, based on the polychorics correlations [66] and using the robust weighted least squares estimation method (WLSMV), wish is robust with non-normal discrete variables [67], with the version 7.4 of MPLUS. Finally, the reliability of the resulting scales was obtained by internal consistency with the full sample.

## Results

### Exploratory analysis

**Fig 1:**
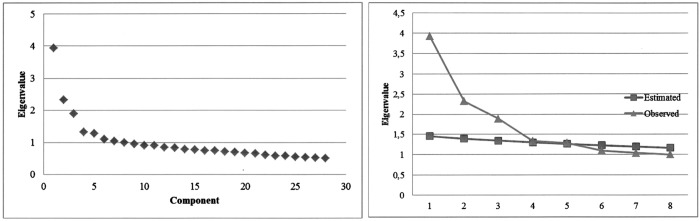
Scree plot (left) and parallel analysis graph (right) for initial factor solution.

An exploratory analysis was conducted with subsample 1. An initial examination indicated the data were suitable for factor analysis techniques (KMO = .806, Bartlett sphericity test χ^2^ = 3942.05, *df* = 378, *p* < .000). The principal component analysis extracted seven factors with eigenvalues greater than 1, which explained 46% of the variance. However, it has been suggested that the criterion of greater than 1 eigenvalue, known as the Kayser-Guttman rule, tends to overestimate the number of factors [64], [68], and graphic analysis of sedimentation, another proposed criterion for determining the number of factors, showed that a three-factor solution may be appropriate (Fig 1). The parallel analysis [69], meanwhile, showed that five factors outweighed the eigenvalue obtained by chance, but only three of them did so with some slack (Fig 1).

Consequently, we decided to force a five factor solution, which explained 38.4% of the variance. Of the five factors, two had all or most of their items with very low loads, so that a solution of three factors, which explained 29.1% of variance, was forced. Two of these factors are correlated (*r* = .23), whereby an oblimin rotation, which does not assume the independence of the factors, was accomplished. By analyzing items of each factor, we found that three of them, one from each factor, still had some difficulty: items 16 and 12 have low relation with their scale, and item 23, "What is true today, will remain true tomorrow" is theoretically inconsistent with the other three items in the factor, which are related to disposition toward authority; therefore, a new EFA without those items was carried out. Thus, three factors were structured: the first grouped nine items corresponding to the factors of Innate Ability (learning control) and Fast Learning (learning speed) of the original EBI; the second included three items corresponding to the factor called Omniscient Authority (source of knowledge) on the EBI; and the third consisted of five items that correspond to the Certain Knowledge factors (certainty of knowledge) and Simple Knowledge factors (knowledge structure) of the EBI. These three factors explain 37.5% of the total variance, and although they show significant correlations, these are small (r < .13; [70], so that the VARIMAX rotation was carried out; Table 2 shows the factor loadings and consistency of the dimensions for this rotated solution.

### Confirmatory analysis

The model achieved in the exploratory phase with subsample 1 was subjected to CFA with subsample 2, which included 40% of all participants. Since the exploratory phase solution was inconsistent regarding the covariance between factors, both solutions were contrasted. The adjustment indicators of both models and the two five factors models in [20], [34], are shown in Table 3.

**Table 3:** Fit indices for models in CFA.

Model	NPar	χ ^2^	DF	P	RMSEA	CI 90%		SRMR	CFI	TLI
						LO	HI			
1	88	446.44	116	.000	.062	.056	.068	1.465	.867	.844
2	85	487.290	119	.000	.064	.059	.070	1.677	.851	.830
3	80	483.262	67	.000	.091	.084	.099	1.75**7**	.723	.624
4	115	889.380	179	.000	.073	.068	.078	1.744	.773	.734

Notes: Model 1, three factors, factors 1 and 3 related. Model 2, three independent factors. Model 3 original Schraw, Bendixen and Dunkle five factors model. Model 4 Mejía and Palma five factor model. NPar, number of parameters in estimation; χ ^2^, chi square; DF, degrees of freedom; p, probability level; RMSEA, root mean square error of approximation; CI, confidence interval; SRMR, standardized root mean square residual; CFI, comparative fit index; TLI, Tucker-Lewis index; AIC, Akaike Information Criterion.

As shown, only the proposed three factor models have good indicators of absolute fit (RMSEA <≈ 0.6; [71]), especially the model with covariated factors, but the two five factor models show an unacceptable fit levels (RMSEA > 0.6; [71]). Incremental indicators do not meet the criteria suggested (TLI > .95, CFI > .95, [62]), but this can be explained by the magnitude of the inter-item correlations that were moderate or low [70], so the null model is poor compared to providing a good baseline adjustment. Also, some authors [72], [73] have argued that an assessment of fit must be performed with absolute fit indicators based on χ^2^.

Although both three factor models may be considered acceptable according to their absolute fit indicators, the compared SRMR suggests that model 1, including covariation between factors 1 and 3, would be slightly better than model 2, without covariance, which is consistent with the indicators of absolute fit. Therefore, model 1 standardized factorial loadings are presented (see Table 4) and was used in the next step: conducting multi-group analyses to determine the factorial invariance model.

**Table 4:** Standardized factorial loadings for model 1.

Item	Factor			Item	Factor		
	1	2	3		1	2	3
1			.480	15	.646		
2			.462	17			.311
3	.539			20	.495		
4		.697		22			.327
5	.594			24	.525		
8	.444			25		.568	
9	.311			26		.594	
11			.515	27	.513		
14	.614						

Multi-group analyses, with the differential of χ^2^ from the baseline free estimated model for both groups, were carried out with subsample 2 subdivided into groups according to the following criteria: random division into halves (n_1_ = 375 and n_2_ = 371, respectively); by gender (male, n = 356, women, n = 369); and city of residence (Arica, n = 358, Iquique, n = 388). The results of these comparisons are shown in Table 5.

**Table 5:** Factor invariance for three factor structure in subgroups defined for three criterions.

Criterion for subgroups	Model	χ^2^	DF	p	P(Δχ^2^ = 0) *
Randomized	Configural	1115.39	232	.000	
	Scalar	1137.21	294	.000	.495
	Metric	1112.20	246	.000	.311
Gender	Configural	1065.73	232	.000	
	Scalar	1142.42	294	.000	.000
	Metric	1082.09	246	.000	.006
City of residence	Configural	1089.95	232	.000	
	Scalar	1242.43	294	.000	.000
	Metric	1129.51	246	.000	.000

*Against configural.

As shown, the model has full factorial invariance between randomly defined subgroups, but not compared by gender and city of residence.

Finally, internal consistency was obtained for each of the three dimensions with the entire sample, with the following indicators: factor 1 α = .73, n = 9; factor 2 α = .62, n = 3; and factor 3 α = .47, n = 5.

## Discussion

The model of epistemic beliefs proposed in [18] has proven difficult to conduct factorial validation. Neither the EQ [17] nor the EBI [20] have shown factorial consistency across different studies, and their reliability has been, in most cases, barely satisfactory. It has been observed that many studies using these instruments have little methodological strength, mainly due to their small sample sizes [23], which could favor these poor results; although recently, some studies have overcome this limitation without obtaining better results [35]. This case has not been different. Using the EBI, the proposed structure of five factors is again not confirmed. Instead, a three-factor structure emerges in exploratory analysis, which is confirmed with proper techniques; and the three-factor structure also presents invariance among subgroups defined by chance, but it not be the case when subgroups are defined by structural variables as gender or residence.

Factors that emerge, however, are not incompatible with those proposed in [18] and restated in [20]. The items in the first factor correspond to two of the original factors in the EBI model: control of learning and learning speed (innate ability and quick learning). Hofer and Pintrich [22] have pointed out that the first of them, learning control or innate ability, corresponds approximately to the idea of understanding intelligence as a fixed entity or as an incremental ability proposed by others [74], [75]; although it has been shown empirically that these two constructs are only moderately correlated and cannot be considered equivalent [24]. The speed of learning or quick learning factor, in turn, is rather a general expectation about learning than a belief about knowledge itself, as noted in [22]. Hofer and Pintrich [22] also note that it would be reasonable that beliefs about control and speed of learning relate to each other, which in fact occur; it could be argued that these two beliefs do not correspond to actual epistemic beliefs but are beliefs about learning, which has been explicitly assumed by Schommer-Aikins [76].

The third factor in the structure emerged in our data also groups items from two dimensions of the original model: stability of knowledge (true knowledge) and structure of knowledge (simple knowledge), what also has been found in other groups [33]. These two dimensions, with source of knowledge (omniscient authority), correspond to the dimensions that would be properly epistemic; however, the three items in the model that correspond to the original dimension source of knowledge, are not grouped with the items corresponding to the dimensions of stability and structure of knowledge in our data, but constitute a separate factor. A content analysis of these three items reveals that this makes sense, since none of the three are related to the actual source or origin of knowledge, but with disposition toward authority: "People should always obey the law",” When someone in authority tells me what to do, I usually do it" and "People should not question authority”. So, probably this group of items that constitute the second factor, rather than reflecting epistemic beliefs, relates to beliefs about the relationship with authority in general and a certain moral disposition toward obedience, as have been suggested [29]. Thus, the factor three appears the one that is properly epistemic, including items of two of the original epistemic dimensions, structure of knowledge and stability of knowledge. This two epistemic dimensions have been found mixed [33] or splitted [53], even mixed with beliefs about learning [32] in other studies with the EBI. It could be for more than one reason. One of them is that in different studies, different sets of items have been retained; so, it could be thought that this factor could split into two factors if other items would be included. But, considering this set of items, the third factor appears to be a more general factor, related broadly with beliefs about knowledge, including items of the two original dimensions, the structure and the stability of knowledge. This interpretation is supported by statistical data in our sample, because communalities are very similar between the items of the third factor, and the difference of communalities between factors are moderate or large, greater than .3. Considered in this way, the three factors that emerge are theoretically consistent, with only one properly epistemic, while others refer to non-epistemic beliefs or dispositions, toward learning or toward obedience to authority.

Whether beliefs about learning should be considered epistemological or not is a longstanding affair, as Leal-Soto [77] discuss. Some authors [22], [78], argue for clarity and consider that only the beliefs related to the nature of the knowledge and the nature of the process of knowing can be considered properly epistemic and must be clearly differentiated of beliefs about learning or other related beliefs; other authors [18], [79], while acknowledging that beliefs about learning are not strictly epistemic, suggest that they should be considered together. Clearly, the factor structure confirmed does not match the model proposed to the EBI nor to the original EQ. Instead, it approaches the structure subsequently proposed by Schommer-Aikins [76] in her embedded systemic model of epistemological beliefs, which takes charge of the aforementioned distinction between properly epistemic beliefs and related beliefs. In this new proposal, she separates beliefs about knowledge (properly epistemic) of those beliefs about learning; and adds a third set of beliefs, those regarding the ways of knowing. Ways of knowing were proposed to describe two approaches to gender-related knowledge [80]. The first, attributed to femininity, is to empathize -to take the position that acts as a source of knowledge to understand the point of view before coming to a critical examination, and it was called connected knowing. In contrast, the second approach was attributed to masculinity and is characterized to start with critical examination, even confrontational with the position that acts as counterparty in knowledge; this way of knowing was called separate knowing. In the embedded systemic model [76], these approaches, which have more to do with relationships with others than with the knowledge, interact with both, beliefs about knowledge and beliefs about learning, influencing self-regulated learning and performance [6], [81]. Although Schommer-Aikins [76] refers only to these two ways of knowing, and keeps the dimension source of knowledge (omniscient authority) linked to the group of epistemic beliefs themselves, it is controversial that the three items that comprise the second factor actually correspond to beliefs about the source of knowledge; rather, as has been raised, these items may reflect certain dispositions toward authority, a subject that could be likened to the third set of beliefs about the ways of knowing that emphasizes the relationship with others in the form of approach to knowledge. We think that this disposition toward authority could complement these ways of knowing, being a broader dimension, which takes over the relational aspects involved in knowledge, incorporating connected and separate modes with the disposition toward authority. Seen this way, this triad—beliefs about knowledge, beliefs about learning, beliefs about the relational dimension involved in learning—approaches what Schraw and Olafson named the epistemological perspective of the world [82]: "a set of beliefs that collectively define one’s attitudes about the nature and acquisition of knowledge" ([83], p.244), and which includes not only belief but also attitudes or dispositions [31].

An alternative interpretation of second factor could be found in Bromme, Kienhues and Porsch [84]. They argued that knowledge is distributed and used differentially. Much of the knowledge we need to solve daily issues are very specialized and specific, so only experts can manage it, and laypersons must know who are the experts in the matter and evaluate them as sources of knowledge, not the knowledge itself; they call this the *second-hand evaluation*, in contraposition to the *first-hand evaluation* of the relevance and veracity of the knowledge itself. We agree with this approach, but the items in the second factor point more clearly to the relational or even moral dimension involved; so it seem us the interpretation of second factor in terms of moral disposition toward authority as a relational factor in the approach to the source of knowledge is more precise in this case.

While the model achieves appropriate adjustment criteria with the data, as shown by the absolute fit indicators, and is theoretically interpretable, the fact that none of the resulting scales achieved satisfactory internal consistency indicators persists. In relation to these indicators, it is noteworthy that the most consistent scale is that which refers to beliefs about the acquisition of learning (learning speed and control of learning), while the less consistent is the scale referring to epistemic beliefs (structure and stability of knowledge). The low reliability of this third factor could be attributed, at first glance, to that its items come from two of the dimensions initially proposed. Nevertheless, these two factors that could be considered epistemic properly have been grouped in different ways in different studies, as we noted previously. For example, Nussbaum and Bendixen [53] report them as two different factors, whereas Muller et al. [33] report them as a single factor. On the other hand, the invariance of the proposed structure between random groups allows us to support the interpretation of the epistemic factor as a single factor, despite its reduced internal consistency. Possibly, this could be improved by modifying some of its items or adding new ones.

The clue of the factorial invariance of this structure in groups by chance but not by gender or residence adds information that, until now, had been neglected. As authors have pointed out, studies that compared epistemic beliefs between groups tended to report only differences between the dimensions, but they do not provide information on whether the factorial structure remains between the groups compared [85]. The absence of invariance between groups by structural variables observed in this study support the claim of Schraw [31] that factor structure of measurement instruments of epistemic beliefs are very dependents of the sample. The lack of consideration of any differences in the factor structure could explain the contradictory results reported, for example, regarding the influence of gender [23].

## Conclusions

Together, this evidence questions the usefulness of the EBI to properly address such epistemic beliefs; rather, it could be considered to provide an approach to the evaluation of the embedded systemic model of epistemic beliefs proposed by Schommer-Aikins [76], incorporating the distinction between beliefs about knowledge and beliefs about learning, and distinguishing the relational dimension, that in the case of the EBI, assimilates to disposition toward authority, which can be understood as a relational factor that influences the second-hand evaluation proposed by Bromme, Kienhues and Porsch [84]. This fact has theoretical relevance, since it provides evidence that supports Schommer-Aikins' reformulation of his model, moving from the schema of epistemological beliefs that include beliefs about learning, to a model that distinguishes the strictly epistemological beliefs from beliefs about learning and other beliefs such as those about the relational dimension involved in evaluating knowledge from sources external to the subject, highlighted by [84]. Regarding epistemic beliefs themselves, we would apply the comment of Bråten et al. that dimensions of epistemic beliefs may be conceptually clear and convincing, but empirically difficult to separate [86], as shown in the third factor of the validated structure; but, since a limitation of our study was to consider strictly the 28 items proposed by Schraw, Bendixen and Dunkle [20], the question of whether epistemological beliefs are best represented unitarily or by distinguishing the dimensions structure and stability still needs to be determined.

From the practical point of view, the poor quality of the indicators obtained (particularly the reliabilities and the proportion of variance explained by the factors) as well as the mismatch of the proposed structure with others reported in the literature, is a new warning about the stability of the instrument and the model, even considering the epistemic beliefs grouped in a single dimension in this instrument. Hence, if the EBI will be used to establish relationships between personal epistemology and other variables, especially when used in different cultural contexts or populations, as Latin-American or Spanish-speaking populations, it is important that the authors clearly specify the factors or dimensions used and the items that were included in each, and the ability of the factor structure to account for variability; otherwise, it will remain difficult to evaluate the results or to make comparisons or generalizations between results from different studies, which constitutes a major obstacle to the advancement of research on this model.

## Supporting information

S1 Table Rotated factor matrix attained with GLS extraction method. (DOCX)

S1 File Data set. (SAV)
