# Supplementary material for: Three-factor structure for Epistemic Belief Inventory: A cross-validation study
Source: PLoS One. 2017 Mar 9;12(3):e0173295. doi: 10.1371/journal.pone.0173295 (PMC5344394; doi:10.1371/journal.pone.0173295)
Supplement: S1 Table — (DOCX) [file pone.0173295.s001.docx]

| Rotated factor matrix (Items are numbered in correspondence with those in Table 2) | | | | | | |  |
| --- | --- | --- | --- | --- | --- | --- | --- |
|  | Factor | | | | | |  |
|  | 1 | | 2 | | 3 | |  |
| Item 1 | | ,104 | | ,192 | | **,304** | |
| Item 2 | | ,024 | | ,028 | | **,421** | |
| Item 3 | | **,458** | | ,263 | | ,054 | |
| Item 4 | | ,026 | | **,687** | | ,007 | |
| Item 5 | | **,542** | | ,063 | | -,022 | |
| Item 8 | | **,403** | | ,054 | | ,067 | |
| Item 9 | | **,298** | | -,056 | | ,197 | |
| Item 11 | | ,028 | | ,070 | | **,488** | |
| Item 14 | | **,553** | | ,109 | | ,106 | |
| Item 15 | | **,567** | | ,043 | | ,044 | |
| Item 17 | | ,055 | | -,089 | | **,365** | |
| Item 20 | | **,470** | | -,035 | | -,113 | |
| Item 22 | | -,082 | | ,058 | | **,359** | |
| Item 24 | | **,482** | | ,029 | | ,052 | |
| Item 25 | | -,029 | | **,590** | | ,116 | |
| Item 26 | | ,143 | | **,484** | | -,009 | |
| Item 27 | | **,508** | | -,041 | | -,093 | |
| Extraction method: GLS  Rotation method: Varimax with Kaiser. | | | | | | |  |
| a. Rotation converged in 4 iterations. | | | | | | |  |
